# Fractographic Analysis of Brinar 400 and Brinar 500 Steels in Impact Testing

**DOI:** 10.1155/2018/2524735

**Published:** 2018-04-29

**Authors:** Beata Białobrzeska, Łukasz Konat, Robert Jasiński

**Affiliations:** Department of Materials Science, Welding and Strength of Materials, Wrocław University of Technology, Wrocław, Poland

## Abstract

Properties of low-alloy boron-containing steels Brinar 400 and Brinar 500 in as-delivered and normalized conditions are considered. Charpy tests carried out within temperature ranges of ductile-to-brittle transition were followed by fractographic analysis. The tests were carried out on specimens with their axes parallel and perpendicular to hot-working direction, at −40°C, −20°C, 0°C, and +20°C. The determined impact properties of Brinar steels were complemented with fractographic analysis performed with use of a scanning electron microscope. It was found that temperatures of ductile-brittle transition were significantly different for the materials in as-delivered and normalized conditions. In addition the tensile tests were carried out, determining basic strength properties of the analyzed materials.

## 1. Introduction

The examinations were performed on selected grades of low-alloy martensitic steels containing boron, with higher resistance to abrasive wear. These steels are more and more often used for working machine parts and their feature especially emphasised by the users is combination of high abrasion resistance with high impact strength—even at lower temperatures. It is worth stressing that these properties are reached, even if chemical composition basically does not include additive elements (except boron) in the amounts commonly accepted as alloying percentages. This is why, from the viewpoint of chemical composition, properties of these steels should not be significantly different from those of unalloyed low- and medium-carbon steels. Nevertheless, presence of boron and selected microadditions, combined with complex operations of thermomechanical treatment, made it possible to obtain highly favorable mechanical properties, unattainable in unalloyed carbon steels. So, these materials should be treated as a separate material group subjected to separate research procedures. In particular, this concerns the problems—often announced by the users—of breaking constructional joints subjected to thermal effects, for example, those caused by welding processes. In service conditions, it is often not considered that these steels are susceptible to such thermal processes, which can lead to degradation of their properties and, especially, reduce their resistance to dynamic loads. In the available scientific literature, there is no sufficient information about influence of variable thermal treatments on properties of these steels. Some results that can be found for selected grades of these materials do not have the nature of a concise and complete model study. For example, in the steels Hardox 400 and Hardox 500, the materials with chemical compositions very close to compositions of Brinar steels, no reduction of impact strength, and no increase of ductile-brittle transition temperature were observed after normalizing [1]. For the presented material group, as well as for other materials with similar usable properties (e.g., steel B27), a drop of impact strength below the accepted brittleness criterion (35 J/cm^2^ [2]) was noted after normalizing—even at temperatures above 0°C [3]. Since more and more steel plants extend their offers by martensitic steels with additions of boron, it seems well grounded to continue research works considering new grades of this type steel. With use of advanced manufacturing technologies, modern low- and medium-carbon steels reach very good mechanical properties for moderate prices. Properties of these steels are decided by their structure obtained after a specific thermal or thermomechanical treatment. In order to obtain high mechanical parameters and abrasion resistance, such typical heat treatment is quenching and low tempering. However, it happens that these steels are delivered by the manufacturers in different conditions, sometimes not including hardening, with a suggestion that heat treatment is to be carried out by the customer. In addition, these steels are most often joined by traditional welding methods, which results in wide structural changes in the entire heat-affected zone, including also the structures characteristic for normalizing, commonly accepted as positive ones.

The above-mentioned structural changes are very clearly observed in steels Brinar 400 and Brinar 500. For this reason, these steels determined by their manufacturer (Ilsenburger Grobblech GmbH) as low-alloy martensitic boron-containing high abrasion-resistant steels were subjected to laboratory examinations. First of all, the research was focused on determining basic mechanical and impact parameters in both as-delivered condition and after normalizing. In addition, impact tests were complemented with detailed fractographic analysis. The accepted research procedure is justified by the fact that, generally, fractures of metals and alloys are never completely of ductile or cleavage nature, since traces of plastic deformation are observed on fracture surfaces even at temperatures close to absolute zero [4, 5]. Therefore, it can be assumed that, at the initial phase of stress action, metal deforms plastically till the moment when strengthening caused by work-hardening creates conditions for cleavage cracking. Even in the cases of very small plastic deformation, impact strength of the material can be relatively high, if the fracture surface is relatively well developed. In such a case, a significant part of energy is absorbed during cracking as surface energy [6].

## 2. Material and Methodology

Test specimens were prepared from metal sheets of Brinar 400 and Brinar 500, 6 mm and 12 mm thick, in as-delivered condition (directly from the steel plant). The specimens were cut out by high-energy water-jet and by spark-erosion, with specimen axes parallel and perpendicular to hot-working direction. A portion of the specimens were normalized by austenitizing at 930°C for 60 minutes followed by air cooling till the ambient temperature. During heat treatment, protective atmosphere of 99.995% argon was applied. Basic mechanical properties of the examined materials, on the grounds of the manufacturer's data, are given in Table 1.

Chemical composition was determined spectrally using a glow discharge analyzer LECO GDS500A, with the parameters *U* = 1250 V and *I* = 45 mA, under 99.999% argon. The results being arithmetic averages of at least five measurements are compared with the manufacturer's data in Table 2.

Brinell hardness measurements of the specimens were made acc. to EN ISO 6506-1:2014-12, using a hardness tester Zwick ZHU with a ball of sintered carbides, under the load of 187.5 kgf. Measurements were made on the specimens previously subjected to microstructure observations. The results are given in Table 1.

Mechanical testing was carried out on proportional rectangular specimens at ambient temperature, acc. to EN ISO 6892-1:2016-09, using a tester MTS 810 equipped with an extensometer with gauge base *L*_0_ = 20 mm. In the tests, constant extension increments were applied up to final fractures of the specimens. Next, ultimate tensile strength (*R*_*m*_), yield strength (*R*_p02_), elongation (*A*_5_), and reduction of area (*Z*) were determined.

Impact energy (KV) and impact strength (KCV) were determined using a Charpy testing machine Zwick-Roell RPK300 at initial energy 300 J, acc. to EN ISO 148-1:2017-02, on specimens with V-notches 2 mm deep. Impact tests were carried out at ambient temperature +20°C, as well as at lower temperatures of 0°C, −20°C, and −40°C. In individual temperature ranges, the specimens were conditioned in a mixture of isopropanol and liquid nitrogen and then transferred to the testing machine within the time not exceeding 5 seconds. Temperature was measured with a thermoelectric sensor type “T” (Cu-CuNi), in accuracy class A (±0.15°C), acc. to EN 60751:2009. The sensor was coupled with a digital temperature meter “Center.” Results of impact testing are given in Section 3.2.

Fractographic analyses and microstructural observations were performed on specimens unetched and etched with 3-% solution of HNO_3_ acc. to PN-H-04503:1961. For visible-light observations, a metallographic microscope Nikon Eclipse MA200 and a stereoscopic microscope Nikon AZ100 were used. For electron microscopy, a scanning electron microscope Jeol JSM-6610A was used at accelerating voltage 20 kV. SEM observations were performed in material contrast using SE detectors.

## 3. Results

### 3.1. Microstructural Analysis

**Figure 1:**
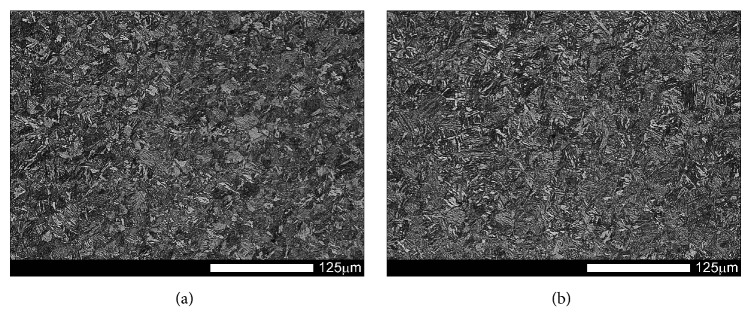
Microstructures of the examined steels in as-delivered condition: (a) Brinar 400 and (b) Brinar 500. Etched with 3-% HNO_3_; light microscopy.

**Figure 2:**
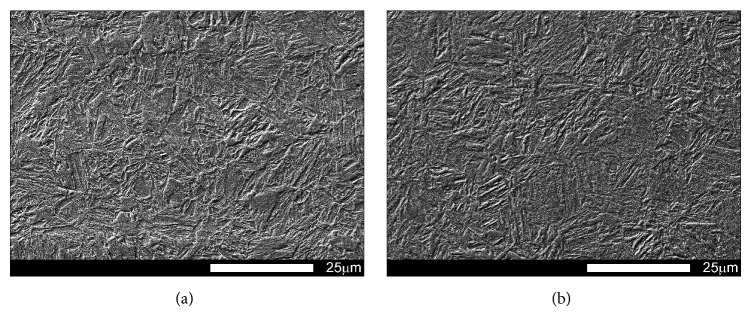
Magnified view of microstructures shown in Figure 1: (a) Brinar 400 and (b) Brinar 500. Etched with 3-% HNO_3_; SEM.

Figures 1 and 2 show microstructures of the examined steels in as-delivered condition. In this condition, the steels have structures of low-carbon, homogeneous martensite with its morphology similar to that of tempered martensite. Moreover, a three-level structure typical for martensite occurring in low-carbon steels can be observed, composed of laths, blocks, and packages. Martensite laths creating a block show identical crystallographic orientation and thus represent the same variant of the created martensite structure.

**Figure 3:**
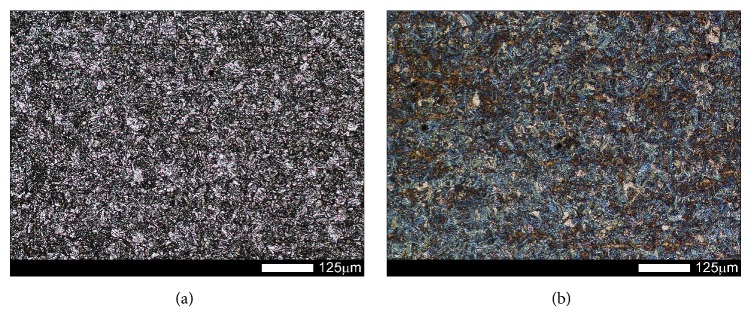
Microstructures of the examined steels in normalized condition: (a) Brinar 400 and (b) Brinar 500. Etched with 3-% HNO_3_; light microscopy.

**Figure 4:**
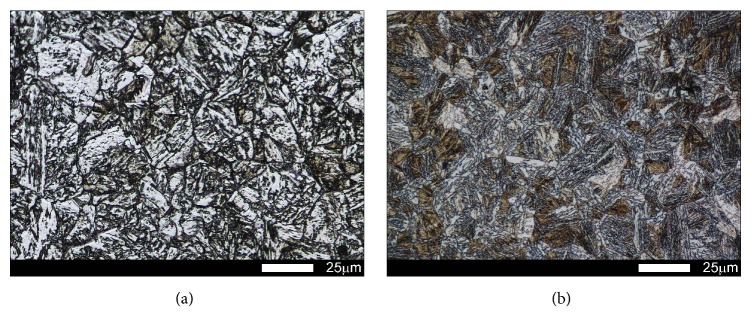
Magnified view of microstructures shown in Figure 3: (a) Brinar 400 and (b) Brinar 500. Etched with 3-% HNO_3_; light microscopy.

In the examined steels, normalization caused variable structural changes. Microstructure of Brinar 400 (Figure 3(a)) was composed mostly of granular and, locally, acicular ferrite. Moreover, sparse precipitates of carbide phases (mostly cementite) were observed; see Figure 4(a). Such a microstructure made it possible to reach hardness of 209 HBW. In the case of Brinar 500, normalization caused extensive structural changes (Figure 3(b)). Its microstructure was composed mostly of acicular martensite (with precipitates of carbide phases) and martensite. Moreover, small amounts of granular ferrite and upper bainite were observed; see Figure 4(b). Irrespective of diversification of Brinar 500 microstructure, normalization led to precipitation of large numbers of carbide phases in the whole material volume. With the observed microstructure, this steel reached hardness of 347 HBW.

**Figure 5:**
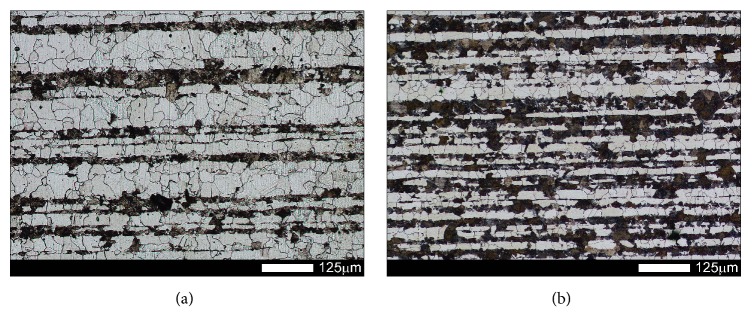
Microstructures of the examined steels in fully annealed condition: (a) Brinar 400 and (b) Brinar 500. Etched with 3-% HNO_3_; light microscopy.

**Figure 6:**
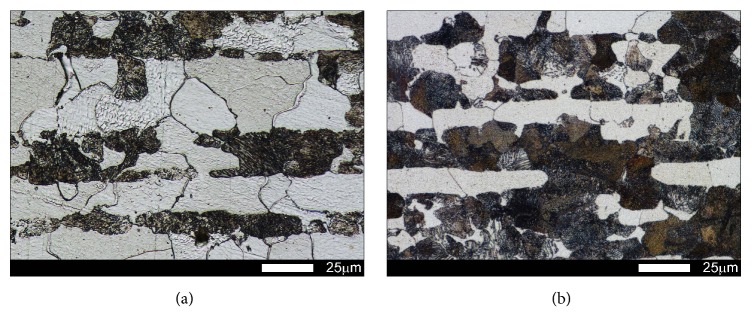
Magnified view of microstructures shown in Figure 5: (a) Brinar 400 and (b) Brinar 500. Etched with 3-% HNO_3_; light microscopy.

Because of high hardenability of both Brinar grades, normalizing of these steels leads to nonequilibrium structures, which significantly hinders identification of their initial condition before any heat-treatment operations. In order to recognise their structural properties, full annealing of both Brinar steels was carried out. Structure of Brinar 400 (Figure 5(a)) after full annealing followed by cooling at 0.1°C/s is composed of banded arrangement of equilibrium ferrite grains with fine-lamellar pearlite. On ferrite grain boundaries, precipitates of tertiary cementite can be clearly observed; see Figure 6(a). Microstructure of Brinar 500 after the same heat treatment (Figure 5(b)) is composed of strongly banded areas of ferrite with fine-lamellar pearlite and sparse colonies of degraded pearlite; see Figure 6(b). Volume percentages of these phases are adequate to carbon concentration in this steel. No precipitates of cementite are observed on ferrite grain boundaries, which suggests that it is completely bounded in the pearlitic mixture.

### 3.2. Results of Impact Testing and Discussion

**Figure 7:**
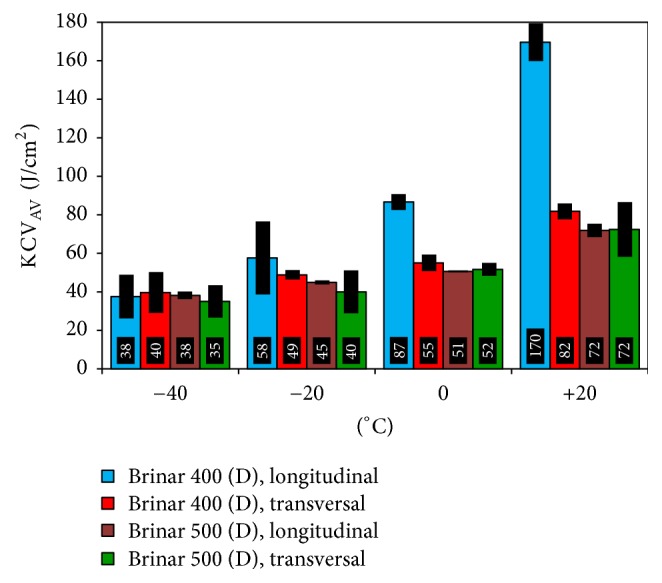
Impact strength of steels Brinar 400 and Brinar 500 in as-delivered condition.

**Figure 8:**
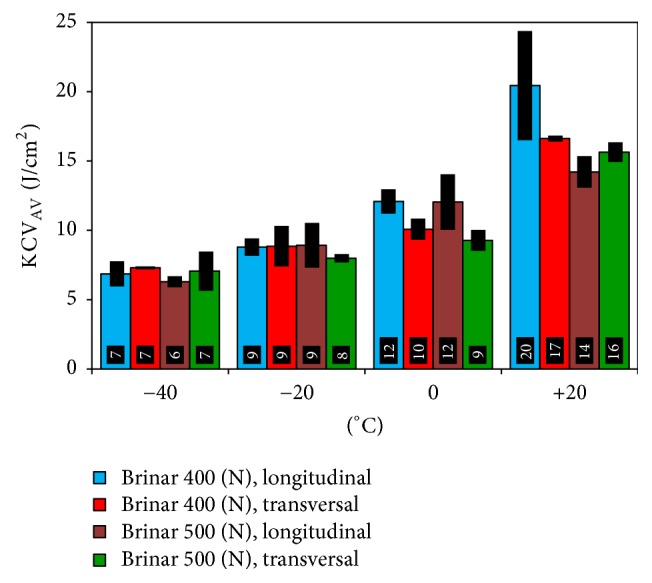
Impact strength of steels Brinar 400 and Brinar 500 in normalized condition.

Results of impact testing of Brinar 400 and Brinar 500 in as-delivered and normalized conditions are shown in Figures 7 and 8. When 35 J/cm^2^ is accepted as the brittleness criterion corresponding to 50-% occurrence of plastic and brittle fractures, it can be said that both analyzed steels meet this criterion in the entire range of testing temperatures; see Figure 7. At all test temperatures (except −40°C), steel Brinar 400 in longitudinal direction showed the highest impact strength. Moreover, important differences between impact strength values in longitudinal and transverse directions were found for this steel. At +20°C, impact strength in longitudinal direction was over 107% higher than that in transverse direction. Smaller differences could be found at 0 and −20°C, when impact strength in longitudinal direction was, respectively, over 57% and 17% higher. At −40°C, impact strength values for all the specimens were similar, while the lowest value equal to the brittleness criterion was found for Brinar 500 in transverse direction.

In normalized condition, impact strength of both Brinar 400 and Brinar 500 at each test temperature was lower than 35 J/cm^2^. Therefore, it could be acknowledged that, in both analyzed steels, mechanical properties were degraded as a result of normalization in comparison to the as-delivered condition. So, it should be considered that high-temperature thermal effects—for example, during welding operations—could lead to brittleness of Brinar 400 and Brinar 500, even at temperatures above 0°C.

### 3.3. Fractographic Analysis

**Figure 9:**
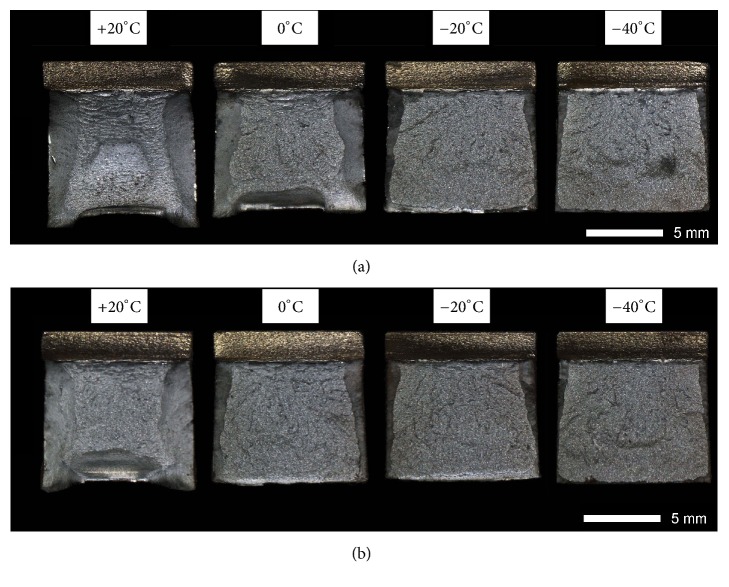
Fracture surfaces of Brinar 400 specimens in as-delivered condition: (a) longitudinal and (b) transverse. Unetched; stereoscopic microscopy.

**Figure 10:**
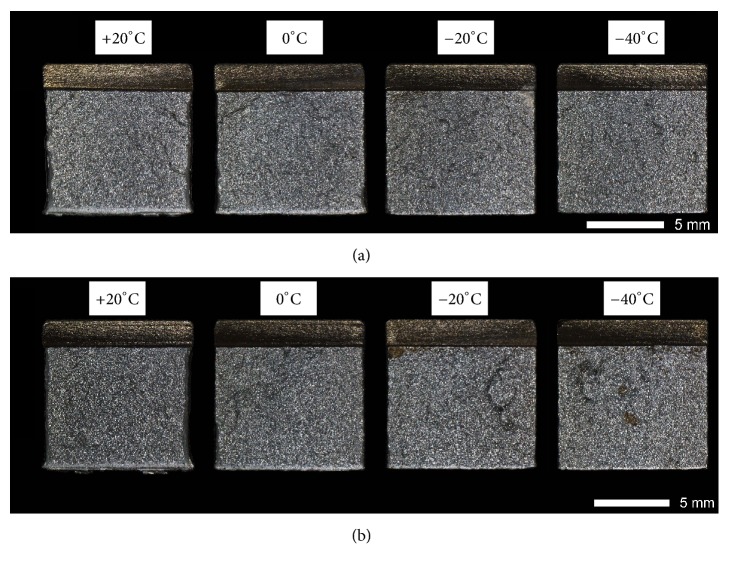
Fracture surfaces of Brinar 400 specimens in normalized condition: (a) longitudinal and (b) transverse. Unetched; stereoscopic microscopy.

**Figure 11:**
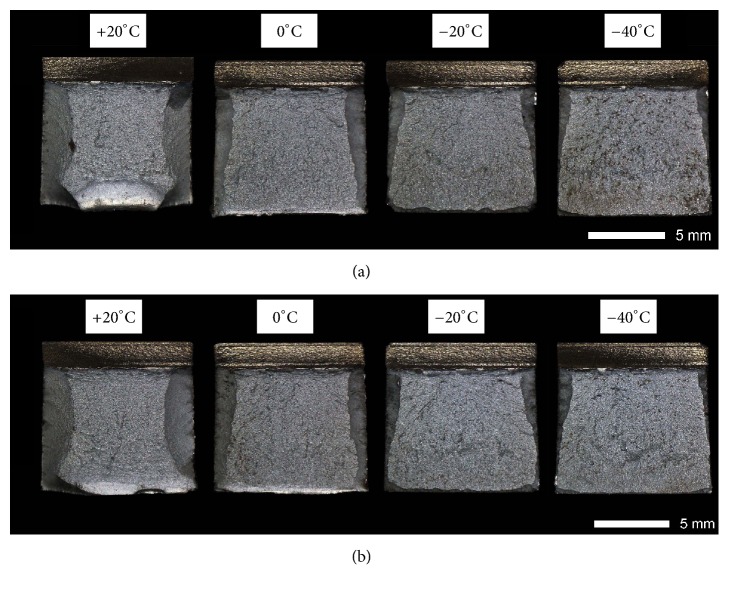
Fracture surfaces of Brinar 500 specimens in as-delivered condition: (a) longitudinal and (b) transverse. Unetched; stereoscopic microscopy.

**Figure 12:**
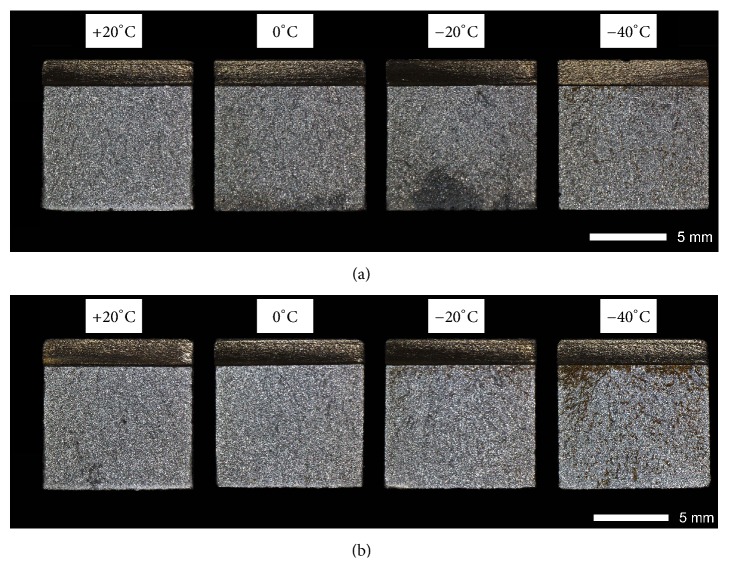
Fracture surfaces of Brinar 400 specimens in normalized condition: (a) longitudinal and (b) transverse. Unetched; stereoscopic microscopy.

Figures 9–12 show macroscopic images of fracture surfaces of Brinar 400 and Brinar 500 specimens after impact testing. On fracture surfaces, percentages of ductile and brittle zones were evaluated, in relation to the accepted brittleness threshold of 35 J/cm^2^. However, it should be indicated that the performed macroscopic analysis revealed some discrepancies with the so defined brittleness criterion.

In specimens of as-delivered Brinar 400 (Figure 9) after impact test at +20°C, percentages of plastic zones on fracture surfaces are significant for both directions: 56% for longitudinal direction and 40% for transverse direction. This made it possible to maintain high impact strength, especially in longitudinal direction. Moreover, large areas of plastic deformations are macroscopically visible on fracture surfaces showing developed topography. Portions of side plastic zones are also significant. After testing at 0°C, percentages of these zones for longitudinal and transverse directions are, respectively, 44% and 18%. After testing at −20°C and −40°C, these percentages for both directions range between 13% and 18%. Nevertheless, even at these negative temperatures, steel Brinar 400 in as-delivered condition maintains high impact strength. This suggests that impact strength level at lower testing temperatures must be affected by nature of cracking in central zone of the fracture surface. Therefore, this zone is the subject of detailed fractographic analysis with use of electron microscopy.

After normalizing, almost 100-% cleavage zones are observed on all fracture surfaces of Brinar 400 specimens (Figure 10), although, after testing at +20°C (especially on surfaces of longitudinal specimens), narrow plastic zones making ca. 10% of the surface can be observed. In comparison to the as-delivered condition, central surface areas of the specimens are more rough and since cracking proceeds through cleavage planes cutting grains, they reflect actual grain size of the broken material. Moreover, no plastic deformation was found by macroscopic observations. Therefore, low impact strength of normalized Brinar 400 is related to missing plastic zones on fractured surfaces and also to structure of the cleavage zone, whose details were revealed by SEM testing.

In as-delivered condition, plastic zones on fracture surfaces of Brinar 500 (Figure 11) after impact testing at ambient temperature make 48% for longitudinal and 35% for transverse direction. Moreover, macroscopic plastic deformation of the fracture is observed. After testing at 0°C, side plastic zones become narrower and make 23% and 21% of fracture surface area, respectively. After impact tests carried out at negative temperatures, plastic zones become even narrower and central cleavage zone constitutes ca. 90% of fracture surface area. Nevertheless, both Brinar 500 and Brinar 400, even at the lowest temperature, show satisfactory impact strength for which, to a large extent, structure of the central zone is responsible.

Fracture surfaces of normalized Brinar 500 (Figure 12) are similar to those of normalized Brinar 400 and, in each case, show absence of macroscopic plastic deformation, as well as absence of plastic zones on sides and under the mechanical notch. Such arrangement is correlated with reduced impact strength in the entire range of testing temperatures.

Due to the large amount of the research material, the microscopic analysis of the fractures of investigated steels was shown only after impact tests carried out at the extreme temperatures of +20°C and −40°C.

#### 3.3.1. Brinar 400: as Delivered

**Figure 13:**
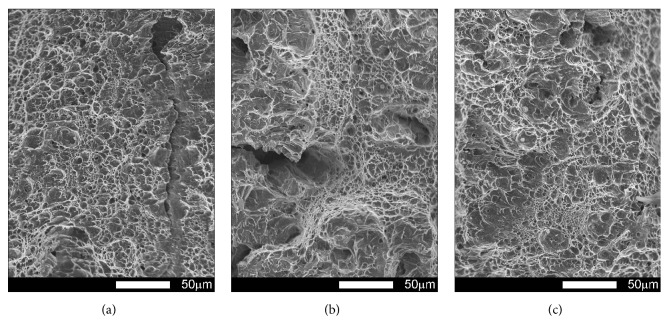
Fracture surfaces of longitudinal Brinar 400 specimens in as-delivered condition after testing at +20°C: (a) under-notch zone; (b) central zone; (c) final-fracture zone. Unetched; SEM.

**Figure 14:**
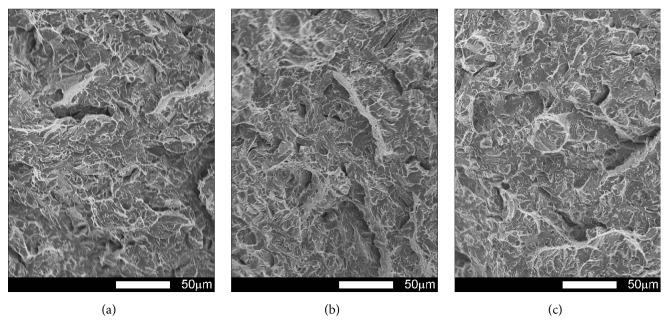
Fracture surfaces of longitudinal Brinar 400 specimens in as-delivered condition after testing at −40°C: (a) under-notch zone; (b) central zone; (c) final-fracture zone. Unetched; SEM.

Fracture surfaces of Brinar 400 specimens in as-delivered condition, cut out in parallel to plastic working direction, are shown in Figures 13 and 14. Macroscopic analysis of fractures after impact tests at +20°C and 0°C showed relatively big parts of plastic zones under mechanical notches and plastic side zones. Microscopic analysis of under-notch plastic zones (Figure 13(a)) revealed that they are ductile fractures with voids of various diameters, partially showing “scaly” structure. It is believed that such a fracture is created as a result of slips followed by decohesion and appearance of microcracks in the planes {100}. Microcracks join each other by shearing their separating walls giving the fracture characteristic appearance of overlapping scales. This indicates that the fracture is initiated by plastic deformation (slip), but cracking itself proceeds basically along determined crystallographic planes [6]. Dimples have parabolic shapes, which proves action of tangent forces in creation of the fracture. Smooth areas with no typical dimpled relief can be also distinguished.

Central zones of fracture surfaces in all the specimens (Figures 13(b) and 14(b)), even those subjected to impact testing at the lowest temperatures, are classified as quasi-cleavage fractures with developed topography and numerous transverse cracks. Such a fracture is typical for steels with martensitic or bainitic structure [2, 6]. This kind of fracture is created by cleavage cracking in small, local areas and next joining the cracks to one cracking plane as a result of plastic deformation. Even if, in this case, the facets are similar to cleavage facets, identification of crystallographic planes is almost impossible because of the presence of “river-like” pattern. It is also impossible to say about a typical quasi-cleavage fracture, because the meandering “river-like” pattern creates, on a large area, dimples whose structure can be similar to that of a ductile fracture. Ridges of quasi-cleavage facets are characterized by developed topography, which also proves plastic deformation during their creation.

The differences observed in structures of central fracture areas can be characterized on the grounds of morphology and portion of cleavage ridges characteristic for a typical ductile fracture. In the specimens tested at negative temperatures, portions of ductile areas inside grains become smaller and cleavage ridges become narrower, while portions of ductile areas in central fracture zones of the specimens tested at ambient temperature are very high. Therefore, it can be ascertained that, in the case of Brinar 400, quantitative changes of impact strength at various test temperatures are correlated with observed features of fracture surfaces, like portions of typically ductile side and under-notch zones, as well as portions of ductile impact ridges in central parts of fracture surfaces.

**Figure 15:**
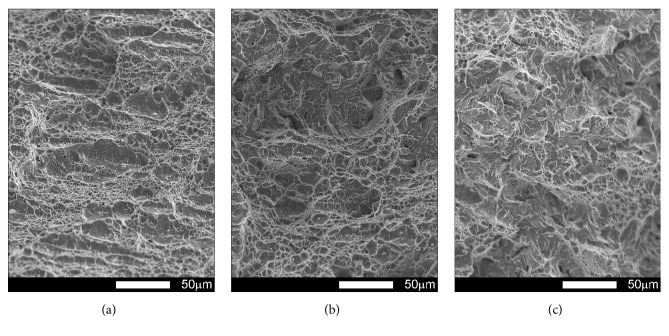
Fracture surfaces of transverse Brinar 400 specimens in as-delivered condition after testing at +20°C: (a) under-notch zone; (b) central zone; (c) final-fracture zone. Unetched; SEM.

**Figure 16:**
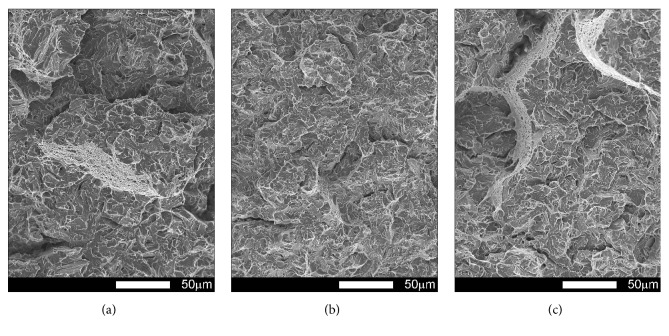
Fracture surfaces of transverse Brinar 400 specimens in as-delivered condition after testing at −40°C: (a) under-notch zone; (b) central zone; (c) final-fracture zone. Unetched; SEM.

Fracture surfaces of Brinar 400 specimens in as-delivered condition, cut out transversely to plastic working direction, are shown in Figures 15 and 16. The performed fractographic analysis showed that fracture surface of a transverse specimen tested at ambient temperature (Figure 15) does not significantly differ from that of a longitudinal specimen. Thus, differences in impact strength values could result mostly from differences in microscopic structures of the specimens. Under the mechanical notch (Figure 15(a)), a plastic zone is visible, with dimples of parabolic shape.

However, central zone (Figure 15(b)) presents a mixed fracture, that is, plastic and quasi-cleavage with developed topography formed in a system of elevations and cavities, showing traces of plastic deformation. Precipitates of intermetallic phases occurring in the dimples of larger diameter acted as stress concentrators [2, 6].

#### 3.3.2. Brinar 500: as Delivered

**Figure 17:**
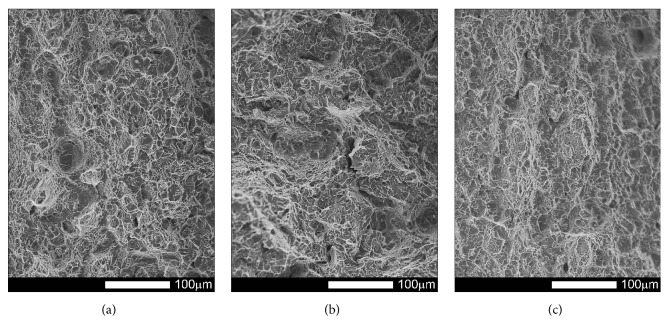
Fracture surfaces of longitudinal Brinar 500 specimens in as-delivered condition after testing at +20°C: (a) under-notch zone; (b) central zone; (c) final-fracture zone. Unetched; SEM.

**Figure 18:**
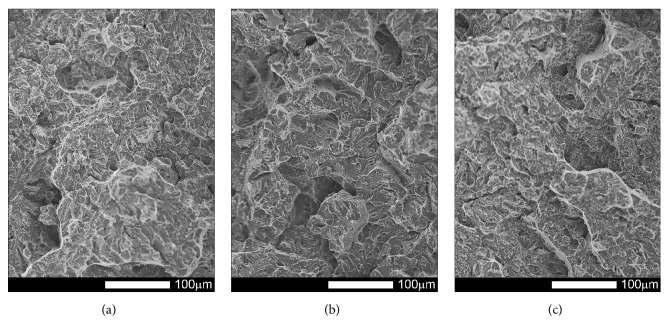
Fracture surfaces of longitudinal Brinar 500 specimens in as-delivered condition after testing at −40°C: (a) under-notch zone; (b) central zone; (c) final-fracture zone. Unetched; SEM.

**Figure 19:**
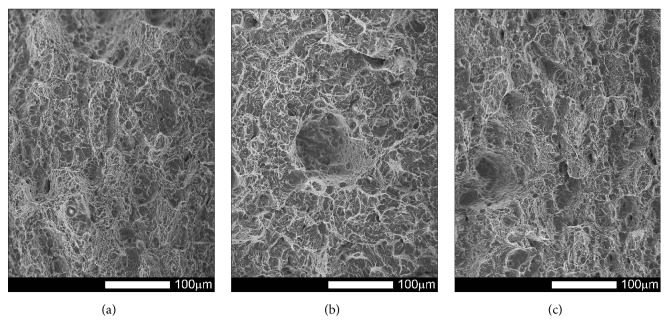
Fracture surfaces of transverse Brinar 500 specimens in as-delivered condition after testing at +20°C: (a) under-notch zone; (b) central zone; (c) final-fracture zone. Unetched; SEM.

**Figure 20:**
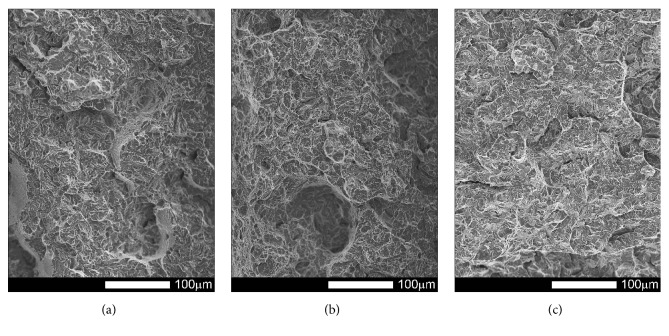
Fracture surfaces of transverse Brinar 500 specimens in as-delivered condition after testing at −40°C: (a) under-notch zone; (b) central zone; (c) final-fracture zone. Unetched; SEM.

Figures 17–20 show microscopic images of individual zones of fracture surfaces of representative Brinar 500 specimens in as-delivered condition. After testing at +20, plastic under-notch zones in longitudinal specimens have dimpled structure; see Figure 17(a). In larger dimples, nonmetallic inclusions or traces after inclusions in form of transverse cracks are visible. Structure of under-notch zone in transverse specimens tested at ambient temperature is similar; see Figure 19(a). Under-notch fracture zones in longitudinal specimens tested at negative temperatures (Figure 18(a)) and in transverse specimens tested at 0°C are developed quasi-cleavage fractures typical for martensitic steels, containing also bands of plastic fractures.

Quasi-cleavage fracture constitutes also the entire central zone of longitudinal and transverse specimens tested within the whole temperature range (Figures 17(b)–20(b)). Topography of the fracture, like in the case of Brinar 400, is still well developed. Occurrence of such topography means that cracking proceeded not in one crystallographic plane, but it jumped from one plane to another by shearing or creating secondary cracks in their separating walls. It was demonstrated that this effect occurs after encountering a screw dislocation, while a jog height is dependent on magnitude of Burgers' vector [6]. Creation of jogs is related to increased energy absorbed during cracking, which is equivalent to reduced brittleness. The jogs change direction of crack propagation. As a result, on some lengths, cracking speed is delayed, which results in bending its front and merging its neighbouring jogs to a “river-like” system. Ridges of these jogs and slopes around transverse cracks make areas of typical plastic fractures that, like in the case of Brinar 400, become smaller at lower test temperature.

Comparison of both Brinar grades indicates that these steels are qualitatively similar. However, plastic fracture areas, especially after testing at ambient temperature, are different (smaller in Brinar 500), which is correlated with differences in impact strength values.

#### 3.3.3. Brinar 400: Normalized

**Figure 21:**
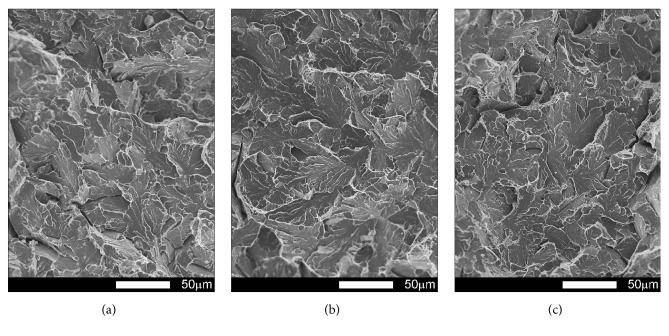
Fracture surfaces of longitudinal Brinar 400 specimens in normalized condition after testing at +20°C: (a) under-notch zone; (b) central zone; (c) final-fracture zone. Unetched; SEM.

**Figure 22:**
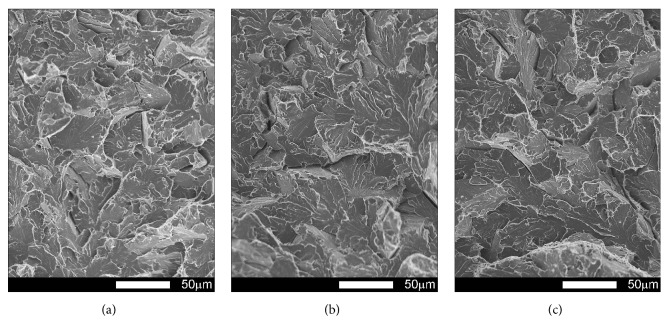
Fracture surfaces of longitudinal Brinar 400 specimens in normalized condition after testing at −40°C: (a) under-notch zone; (b) central zone; (c) final-fracture zone. Unetched; SEM.

**Figure 23:**
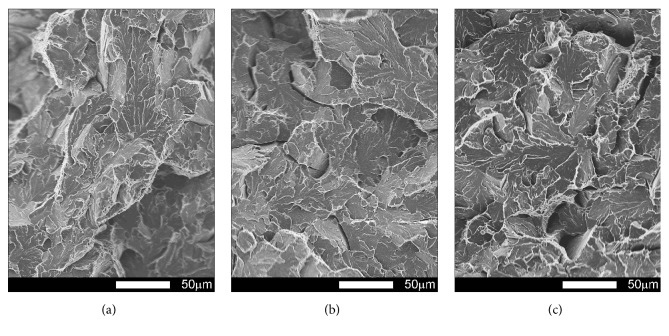
Fracture surfaces of transverse Brinar 400 specimens in normalized condition after testing at +20°C: (a) under-notch zone; (b) central zone; (c) final-fracture zone. Unetched; SEM.

**Figure 24:**
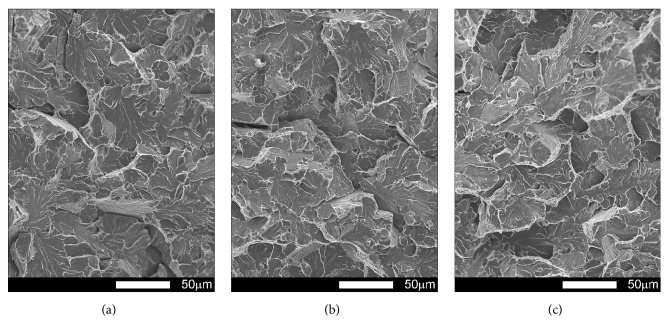
Fracture surfaces of transverse Brinar 400 specimens in normalized condition after testing at −40°C: (a) under-notch zone; (b) central zone; (c) final-fracture zone. Unetched; SEM.

Figures 21–24 show SEM images of representative fracture surfaces of Brinar 400 specimens in normalized condition. Earlier macroscopic examinations of Brinar 400 fractures after normalizing in both longitudinal and transverse specimens tested at +20°C did not reveal any plastic side and under-notch zones. That was correlated with lower impact strength values in normalized condition at ambient temperature and above.

Irrespective of the direction in which a specimen was cut out, central fracture zone is of cleavage nature with characteristic river-like relief; see Figures 21(b) and 23(b). Surface topography in that zone is developed and contains transverse cracks. Absence of side plastic zones and low portion of plastic areas in central part of the fracture are correlated with the fact that, in the examined steel, ductile-brittle transition occurs already at positive temperatures.

Fractures of Brinar 400 specimens tested at lower temperatures are also typical cleavage fractures created by cracking along specific crystallographic planes; see Figures 22(b) and 24(b). Fracture surfaces show jogs with characteristic river-like relief and their presence means that cracking did not proceed in one crystallographic plane but jumped from one plane to another. As Maciejny [6] reports, this effect occurs when the cracking front encounters a screw dislocation and size of the jog is dependent on magnitude of Burgers' vector. Moreover, the jogs influence direction of crack propagation. As a result, the crack is delayed on some lengths and contributes to merging adjacent jogs in a river-like system or a “fan-like” relief. Besides typically cleavage facets, small areas of plastic fracture can be found on the surface. This results from the fact that, in polycrystalline materials, a number of grains exist with their orientation or state of stress unfavorable for creation of a cleavage fracture [9]. A characteristic detail on surfaces of the analyzed fractures is presence of intercrystalline transverse cracks and sharp, flat, jog-free elevations similar to partial tears.

#### 3.3.4. Brinar 500: Normalized

**Figure 25:**
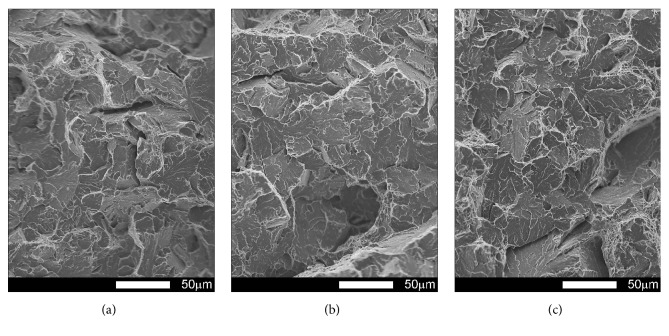
Fracture surfaces of longitudinal Brinar 500 specimens in normalized condition after testing at +20°C: (a) under-notch zone; (b) central zone; (c) final-fracture zone. Unetched; SEM.

**Figure 26:**
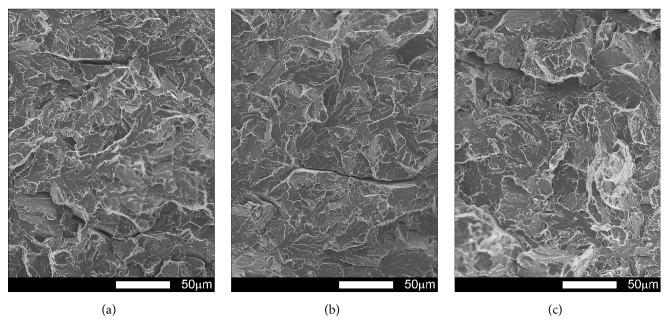
Fracture surfaces of longitudinal Brinar 500 specimens in normalized condition after testing at −40°C: (a) under-notch zone; (b) central zone; (c) final-fracture zone. Unetched; SEM.

**Figure 27:**
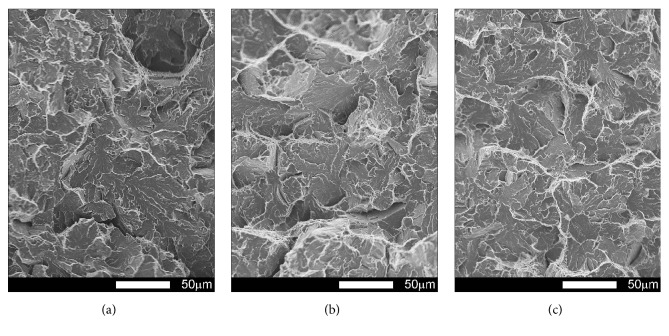
Fracture surfaces of transverse Brinar 500 specimens in normalized condition after testing at +20°C: (a) under-notch zone; (b) central zone; (c) final-fracture zone. Unetched; SEM.

**Figure 28:**
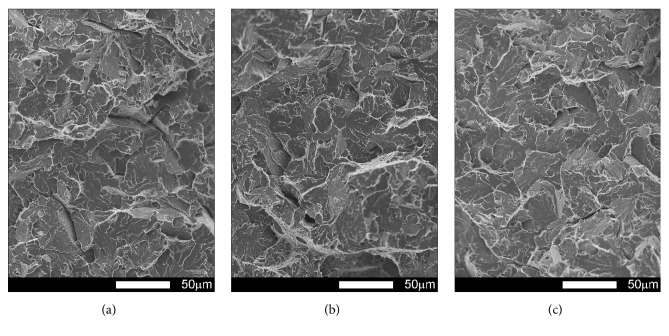
Fracture surfaces of transverse Brinar 500 specimens in normalized condition after testing at −40°C: (a) under-notch zone; (b) central zone; (c) final-fracture zone. Unetched; SEM.

Figures 25–28 show representative fracture surfaces of Brinar 500 specimens in normalized condition. A fracture of normalized Brinar 500, even after testing at +20°C, is a typical cleavage fracture (Figures 25(b) and 27(b)) with characteristic system of “rivers” that combine in “drainage areas” towards expansion direction of the main crack. Presence of the river-like relief indicates additional expenditure of energy during creation of a fracture [2, 6]. Locally, in the places where additional cracks originated on grain boundaries, fan-like areas can be observed.

Moreover, traces of secondary cracks can be seen on surfaces of facets. Apart from cleavage facets, bands of plastic fracture running along cleavage rigs occur on the fracture surface. After impact testing of normalized steel Brinar 500 at lower temperatures (Figures 26(b) and 28(b)), central, quite developed parts of fracture surfaces are cleavage fractures in that plastic areas are smaller than those in normalized Brinar 400. Structure of the observed facets is typical, with “drainage areas” and traces of intercrystalline secondary cracks.

It can be generally said that, in normalized Brinar 500, ductile-brittle transition occurs already at positive temperatures, like in the case of Brinar 400.

The obtained impact testing results, as well as the performed fractographic analyses lead to the conclusion that, during normalization, steels Brinar 400 and Brinar 500 undergo degradation processes manifesting themselves, first of all, by reduced impact energy. Generally, presence of dominating cleavage fracture in structures of the examined steels after normalizing is characteristic for steels with ferritic-pearlitic structure [6]. Presence of pearlite in steel structure is identified with reduced crack toughness [10]. In the analyzed steels in normalized condition, cleavage fractures occurred already after testing at ambient temperature, while, in analogous examinations of steels Hardox 400 and Hardox 500, brittle cracking occurred at negative temperatures only [11–13]. Moreover, at ambient temperature, steels Hardox 400 and Hardox 500 showed impact strength higher than 100 J/cm^2^, maintaining large areas (nearly 50%) of side plastic zones on their fracture surfaces. Central fracture zones in these steels were of typically cleavage nature. At 0°C, impact strength values of Hardox steels were still significantly higher than 35 J/cm^2^ [11–13]. It is also interesting that fractures of Hardox steels in as-delivered condition, tested at ambient temperature, were ductile with dimpled structure [1], while fractures of Brinar steels were mixed—that is, quasi-cleavage with a significant part of ductile fractures. Quasi-cleavage fractures with plastic areas are typical for martensitic steels not subjected to tempering after quenching, whereas structure of tempered martensite results in dimpled ductile fractures [14, 15].

### 3.4. Tensile Tests

The most important differences in the determined basic strength properties apply to both tested steel grades Brinar 400 and Brinar 500 in the case of juxtaposition of materials before and after normalizing annealing. The differences concern, first of all, the strength properties (*R*_p0.2_ and *R*_*m*_), which are reduced in the case of Brinar 400 steel by 44% for *R*_p0.2_ and by 56% for *R*_*m*_ (as-delivered condition and annealed condition). Young's modulus value remains similar for the tested materials, with the exception of the Brinar 500 steel in the normalized state, for which Young's modulus drops by 10% compared to this material in the delivery state.

Steel Brinar 400 in the as-delivered condition is characterized by the most favorable value of the strength ratio *R*_p0.2_/*R*_*m*_ (0.93). Normalization results in a significant decrease in the *R*_p0.2_/*R*_*m*_ ratio for both tested steel grades, amounting to 23% for Brinar 400 steel and 29% for Brinar 500 steel.

On the other hand, the ductility represented by the percentage elongation (*A*_5_) increased as a result of normalizing annealing by 83% for Brinar 400 steel and 103% for Brinar 500 steel. The reduction of area value (*Z*) did not change significantly for the tested materials; however, it remains with the connection with materials' fracture and reflecting the type of the fracture. For Brinar 400 steel (in the as-delivered condition), despite the relatively small average value of elongation (*A*_5_), the slip fracture has plastic features in the macroscopic image, and the reduction of area takes the value of 52%. Only in the case of Brinar 500 steel (in the normalized state) does the value of reduction of area significantly decrease to 33%, corresponding to the character of the brittle fracture, confirming the results of fractographic analysis for this material.

The change of the forming direction of the tested Brinar 400 and 500 steels did not lead to a significant change in their strength and plastic properties.

## 4. Summary

When 35 J/cm^2^ is accepted as the brittleness criterion corresponding to 50% occurrence of plastic and brittle fractures, it can be acknowledged that, in as-delivered condition, the analyzed steels are resistant to brittle cracking in the entire range of test temperatures. In normalized condition, both Brinar 400 and Brinar 500 reached impact strength lower than 35 J/cm^2^ at each test temperature. In this case, ductile-to-brittle transition was noted already at ambient temperature, which can be clearly explained by occurrence of degradation changes resulting from carried-out thermal treatments.

The performed macroscopic analysis of fracture surfaces showed some discrepancies between the qualitative and the quantitative brittleness criteria. In the case of fracture surfaces of as-delivered, both longitudinally and transversely cut-out specimens of Brinar 400 and Brinar 500 after impact testing at +20°C, portions of plastic zones ranged between 35% do 56%, which guaranteed high level of impact strength. Significant portions of plastic zones were maintained at 0°C. However, after impact testing at negative temperatures, central cleavage zones covered nearly 90 and 100% of fracture surface areas. In normalized condition, fracture surfaces always showed absence of macroscopic plastic deformation, as well as absence of side and under-notch plastic zones.

SEM examinations of fracture surfaces of as-delivered Brinar 400 and Brinar 500 showed that central zones of fracture surfaces were of quasi-cleavage nature with developed topography, containing numerous transverse cracks and plastic cleavage ridges, whereas plastic side zones and under-notch zones showed dimpled structure. In larger dimples, nonmetallic inclusions or traces after inclusions in form of transverse cracks were found.

Microscopic observations of the analyzed steels in normalized condition showed typically cleavage fractures with characteristic “river-like” relief, containing small portions of plastic dimpled zones.

## Figures and Tables

**Table 1:** Selected mechanical properties of steels Brinar 400 and Brinar 500: D: as-delivered, N: normalized, MD: manufacturer's data [7, 8], OR: own results, L: longitudinal direction, T: transverse direction, and NA: not available.

Steel grade	*E*		*R*_p0.2_		*R*_*m*_		*A*_5_		*Z*		KCV_−20_		Hardness	
	GPa		MPa		MPa		%		%		J/cm^2^		HBW	
	MD	OR	MD	OR	MD	OR	MD	OR	MD	OR	MD	OR	MD	OR
Brinar 400														
D(L)	NA	2.03 ± 0.06	1100	1156 ± 29	1300	1248 ± 3	12	10.3 ± 0.3	NA	52.2 ± 2.6	35	58 ± 19	360–440	412 ± 2
D(T)	NA	2.04 ± 0.14	NA	1149 ± 41	NA	1242 ± 34	NA	10.0 ± 0.6	NA	50.2 ± 6.5	NA	49 ± 2.4		
N(L)	NA	1.97 ± 0.14	NA	493 ± 7	NA	695 ± 11	NA	18.8 ± 2.1	NA	46.6 ± 3.5	NA	9 ± 0.6	NA	209 ± 2
N(T)	NA	1.92 ± 0.05	NA	485 ± 8	NA	679 ± 7	NA	20.6 ± 1.7	NA	47.0 ± 1.6	NA	9 ± 1.5		
Brinar 500														
D(L)	NA	2.10 ± 0.23	1350	1278 ± 10	1500	1494 ± 8	8	11.0 ± 0.2	NA	46.2 ± 8.6	25	45 ± 1.0	480	472 ± 4
D(T)	NA	1.98 ± 0.12	NA	1279 ± 27	NA	1493 ± 14	NA	10.9 ± 1.0	NA	43.4 ± 2.4	NA	40 ± 11.1		
N(L)	NA	1.88 ± 0.05	NA	560 ± 36	NA	926 ± 6	NA	22.4 ± 1.0	NA	33.3 ± 3.9	NA	9 ± 1.6	NA	347 ± 10
N(T)	NA	1.85 ± 0.03	NA	545 ± 6	NA	937 ± 6	NA	21.7 ± 0.1	NA	33.4 ± 1.7	NA	8 ± 0.3		

**Table 2:** Chemical compositions of steels Brinar 400 and Brinar 500: MD: manufacturer's data [7, 8], OR: own results, and NA: not available.

Element	Brinar 400		Brinar 500	
	Chemical composition [wt%]			
	MD	OR	MD	OR
C	≤0.18	0.20	≤0.28	0.30
Mn	≤2.00	1.13	≤1.50	0.97
Si	≤0.50	0.23	≤0.80	0.60
P	≤0.015	0.012	≤0.020	0.015
S	≤0.005	0.001	≤0.005	0.001
Cr	≤1.55	0.61	≤1.50	0.87
Ni	NA	0.45	NA	0.04
Mo	≤0.60	0.31	≤0.40	0.20
V	NA	0.040	NA	0.005
Cu	NA	0.025	NA	0.020
Al	≤0.10	0.071	≤0.10	0.038
Ti	NA	0.005	NA	0.007
Nb	NA	0.013	NA	0.000
Co	NA	0.012	NA	0.013
B	≤0.005	0.0023	NA	0.0008

## Data Availability

The data used to support the findings of this study are available from the corresponding author upon request.
